# Analyzing the distribution patterns of soybeans and rapeseeds in china under future climate change scenarios utilizing the maxent model

**DOI:** 10.1371/journal.pone.0342400

**Published:** 2026-04-01

**Authors:** Yi Xiang, Zhibo Han, Shan Zhang, Feili Wei

**Affiliations:** 1 Guangxi Key Laboratory of Environmental Processes and Remediation in Ecologically Fragile Regions, Guangxi Normal University, Guilin, China; 2 College of Environment and Resources, Guangxi Normal University, Guilin, China; KGUT: Graduate University of Advanced Technology, IRAN, ISLAMIC REPUBLIC OF

## Abstract

Soybeans and oilseed rape are the main oil crops in China. Assessing the potential geographical distributions of soybean and oilseed rape in China under global warming trends can provide a scientific basis for agricultural production. In this study, 19 climate factors were used as environmental settings based on the MaxEnt model, and the dominant factors were screened and analyzed to predict the potential distributions of soybean and oilseed rape under three climate scenarios of shared socio-economic pathways (SSP) in simulated historical and future periods and to analyze the change trends. The main conclusions were as follows: (1) There are three predominant climatic factors affecting soybeans, among which the mean temperature during the warmest season and the precipitation in the wettest month are the most significant. For rapeseed, five factors are dominant, with annual precipitation and the mean temperature during the coldest season being the primary ones. (2) It is projected that in the next nearly 50 years, the highly suitable cultivation areas for soybean and rapeseed in China will increase by at least 137,000 km² compared to historical periods, representing an increase ranging from 0.14% to 9.33%. A northward shift of cultivation boundaries primarily characterizes this expansion, while most northwest inland areas will become unsuitable for cultivation. (3) In the intermediate stage of the 21st century under the SSP126 scenario, the potential suitable areas for soybeans and rapeseed will have the largest increases of 4.28% and 8.28% respectively, indicating that the planting effect will be the best during this period. The results indicate that future climate conditions will be more suitable for the growth of soybean and rapeseed. Cultivation area selection for soybean and oilseed rape in suitable eastern areas, the selection and breeding of good varieties, and yield improvement to obtain the maximum benefits should be considered.

## 1. Introduction

Since the Industrial Revolution, climate change has directly affected the distribution and yield of crops, threatening global agriculture and food security [1,2]. From 1951 to 2020, surface temperature, precipitation, and the frequency of extreme weather events increased in China’s major agricultural regions, leading to droughts in the north and floods in the south [3]. In 2021, China’s mean surface temperature was 0.97°C higher than the average value, and the rate of warming was higher than the global average. By the end of this century, precipitation in China is predicted to increase in intensity with a slight decrease in frequency, and rainfall is expected to increase in the north and decrease in the south, with more in autumn and winter and less in spring and summer [4]. Zhou [5] and Li [6] evaluated the possible impact and countermeasures of China’s agricultural development against this background. Domestic scholars mainly selected rice [7–10], wheat [11,12] and other crops for potential distribution simulation, indicating that in the future, the planting boundaries of rice and other crops will move north and the growing period will be shortened. It is urgent to take mitigation measures to slow down future migration and maintain the stable development of agriculture.

Both soybeans and rapeseed prefer a warm and humid environment. It has a history of cultivation in China for more than a thousand years and has a relatively high economic value. In recent years, China’s annual consumption of soybeans has been about 110 million tons, but the average output from 2019 to 2021 was only 1961 kg/hm^2^, resulting in year-round dependence on imports [13]. The rapeseed planting area is affected by climate abnormalities and the production is seriously reduced. If the climate changes in the future, it will inevitably affect the yield of soybeans and rapeseed, resulting in an imbalance between supply and demand in the market. Based on the purpose of ensuring food security and optimising the planting layout, the future climate-driven distribution modelling of soybeans and rapeseed is conducive to predicting the future yield potential in advance, reducing planting risks, and providing an important scientific basis for the formulation of medium- and long-term planting plans and policies.

However, the scale of research on the distribution of soybeans and rapeseed is relatively small and usually focuses on a single crop. The factors examined tend to emphasize socioeconomic aspects such as market prices, land resources, and government policies, with limited attention given to the impacts of climate change. And the research methods mainly include mathematical and theoretical models, such as the climate suitability model [14,15], linear regression model [16], and species distribution model [17]. The climate suitability model has made a positive contribution to the quantitative development of agro-meteorology, but based on its limitations in terms of the plant’s mechanism and compensation for climatic elements, it is still necessary to consider the combination of analytical and actual results and basic research on crop meteorology for a more in-depth analysis [18]. In contrast, the MaxEnt model can integrate theoretical assumptions under all constraints, aiming to determine the probability distribution of the maximum entropy value of a species [19,20], and is widely used in fields such as invasive biology and crop planting planning [21]. In addition, the MaxEnt model has the advantages of a small sample size, high precision, good stability, and high compatibility [22]. It can not only solve the problem of China’s lack of comprehensive records of soybean and rapeseed cultivation, but also reduce the difficulty of data collection, reduce the overfitting caused by fluctuations of climate factors in the future, and truly reflect the impact of climate factors. Therefore, the objectives of this study were to (1) select suitable bioclimatic variables for Maxent models, (2) evaluate the potential geographical distributions of soybean and oilseed rape in China, and (3) categorize the levels of planting suitability and employ spatial analysis to estimate the potential suitable area for each level.

## 2. Materials and methods

### 2.1. Sample distribution data

**Fig 1:**
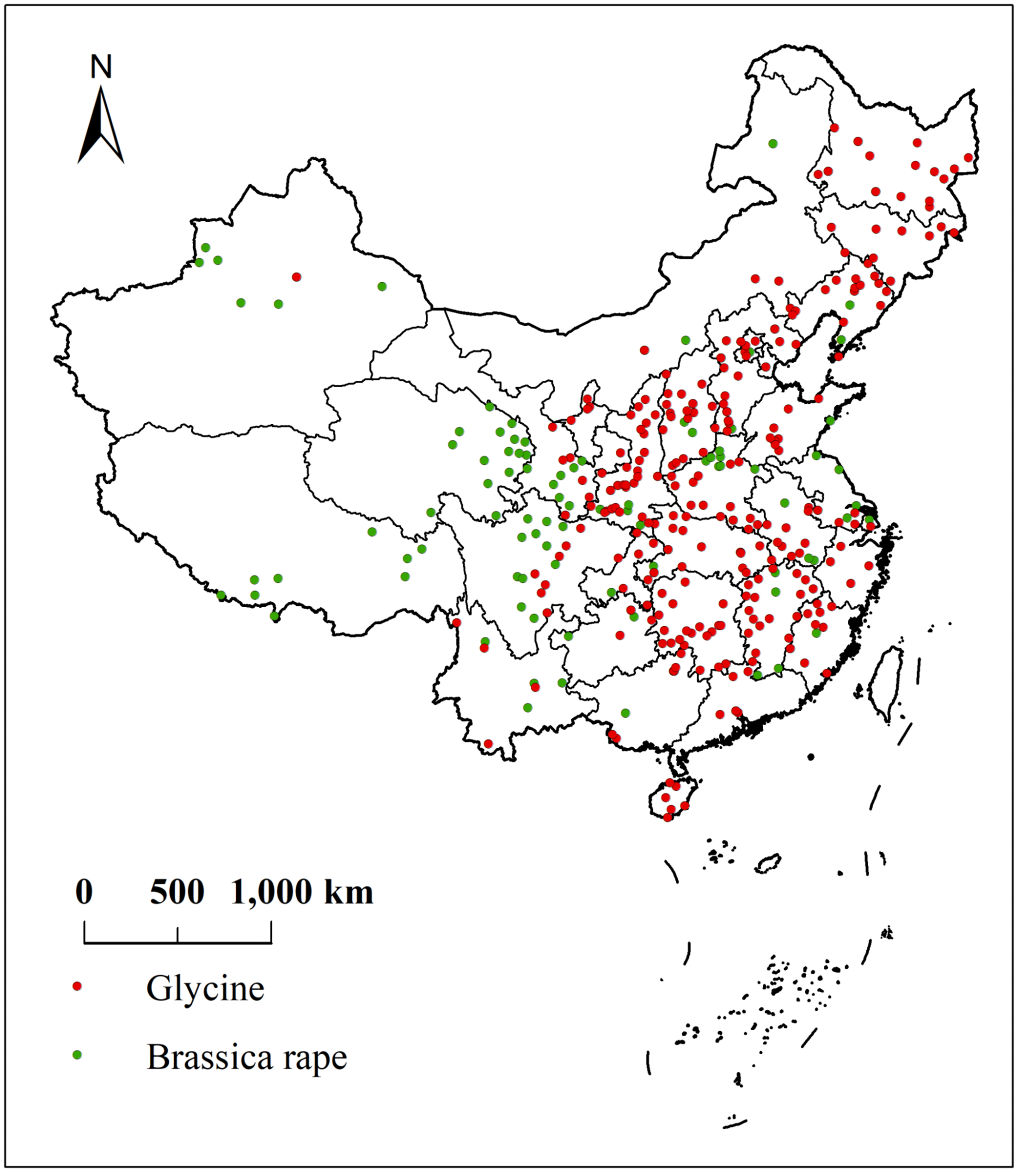
Distribution of Actual Sample Points of the Crop. **(Map Source: Standard Map Service of the Ministry of Natural Resources, China (Approval NO.:GS(2020)4619), and the base map was not modified. (http://bzdt.ch.mnr.gov.cn/**).

The sample data for soybeans and rapeseed were obtained from the Plant Science Data Centre (https://www.plantplus.cn/cn), and included 271 soybeans and 122 rapeseed samples. Using the ENMTools tool, all sample data are spatially filtered, and abnormal distribution points are screened, and the final sample data sets of valid soybeans and rapeseed are 260 and 121. The sample latitude and longitude coordinates were stored in an Excel database in CSV format and used to construct the MaxEnt model, with a training set of 75% and a test set of 25%. Fig 1 shows the actual sample distributions of soybean and oilseed rape.

### 2.2. Meteorological data and scenarios

The climate data used in this study for the historical period (1970–2000), the middle of the 21st century (2041–2060), and the end of the 21st century (2081–2100) were obtained from the World Climate Data website (https://www.worldclim.org/) and mainly included 19 bioclimatic factors (Table 1), with a spatial resolution of 2.5 minutes. Among them, the data for the future period were adopted from the atmospheric circulation model of BCC-CSM2-MR, and the corresponding climate factor data under the three climate scenarios of shared socioeconomic pathways released by the Sixth Assessment Report of the IPCC, namely SSP126 (low forcing), SSP245 (medium forcing), and SSP585 (high forcing), were selected [23].

**Table 1:** Climate factors used in the study.

Abbreviation	Description	Unit
BIO1	Annual Mean Temperature	°C
BIO2	Mean Diurnal Range (Mean of Monthly (Max Temp – Min Temp))	°C
BIO3	Isothermality (BIO2/BIO7) (×100)	°C
BIO4	Temperature Seasonality (Standard Deviation ×100)	°C
BIO5	Max Temperature of Warmest Month	°C
BIO6	Min Temperature of Coldest Month	°C
BIO7	Temperature Annual Range (BIO5-BIO6)	°C
BIO8	Mean Temperature of Wettest Quarter	°C
BIO9	Mean Temperature of Driest Quarter	°C
BIO10	Mean Temperature of Warmest Quarter	°C
BIO11	Mean Temperature of Coldest Quarter	°C
BIO12	Annual Precipitation	mm
BIO13	Precipitation of Wettest Month	mm
BIO14	Precipitation of Driest Month	mm
BIO15	Precipitation Seasonality (Coefficient of Variation)	mm
BIO16	Precipitation of Wettest Quarter	mm
BIO17	Precipitation of Driest Quarter	mm
BIO18	Precipitation of Warmest Quarter	mm
BIO19	Precipitation of Coldest Quarter	mm

In order to screen out the key dominant climate factors and solve the collinearity problem of bioclimatic factors, ENMTools and Maxent are used for correlation analysis. By comparing bioclimatic variables with a correlation close to 1, combined with the contribution table, reasonable biological climate variables can be selected.

### 2.3. Model construction

The area under the curve (AUC) for the receiver operating characteristic (ROC) curve was used to assess model performance, with values ranging from 0 to 1 [24]. The AUC value is generally rated as having substandard (0.5–0.6), poor (0.6–0.7), fair (0.7–0.8), good (0.8–0.9), or excellent (0.9–1.0) predictive accuracy [25]. The random test percentage of the model is 25, the number of replicates is 10, the replication run type is set to Bootstrap, and the maximum iteration is 5,000 times. Finally, do a jackknife to measure variable importance and the response curve, and output it as “Logistic”. Based on the results, the dominant climatic factors affecting the distribution were screened. Under the condition that the model parameters remain unchanged, the dominant climate factors are used to simulate the historical and future distribution of soybeans and rapeseed.

### 2.4. Suitability classification

The model prediction result has a distribution probability value (P) of 0–1 and indicates the climatic suitability of the species in the predicted distribution area, and the larger the P value, the higher the suitability [26]. Combined with the relevant literature and the study of climatic conditions in the study area, the potential geographical distributions of soybean and oilseed rape were classified according to the following criteria: unsuitability (0 < P ≤ 0.05), low suitability (0.05 < P ≤ 0.33), moderate suitability (0.33 < P ≤ 0.66), and high suitability (0.66 < P ≤ 1). Finally, use ArcGIS technology to process the simulation outcomes and regionalization levels, and generate potential contour maps and suitable area sizes for different epochs.

## 3. Results

### 3.1. Model accuracy verification

By screening 19 environmental factors (see the supplementary materials for details), the dominant environmental factors affecting the soybean distribution in China were BIO12 (annual precipitation), BIO5 (max temperature in the warmest month), and BIO16 (precipitation in the wettest quarter). The dominant climatic factors affecting the oilseed rape distribution in China were BIO12 (annual precipitation), BIO11 (mean temperature in the coldest quarter), BIO5 (max temperature in the warmest month), BIO15 (precipitation seasonality), and BIO7 (temperature annual range).

**Fig 2:**
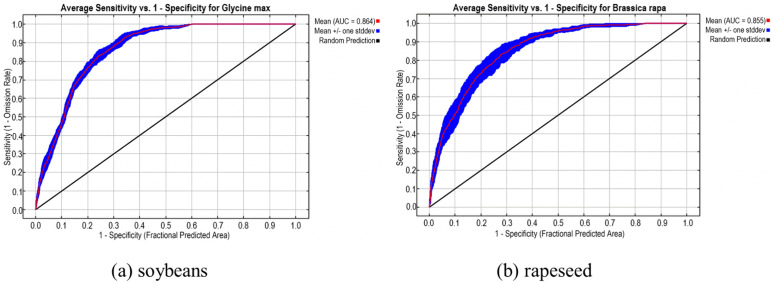
ROC Curve Analysis of Simulated Distribution of Soybeans and Rapeseed under Dominant Climate Factors.

The dominant environmental factors selected for soybean and rapeseed were used as environmental variables. After running the model, the ROC curves obtained are shown in Fig 2. The average AUC value for soybean was 0.864, and for rapeseed, it was 0.855, falling within the good range (0.8 ~ 0.9), indicating that these dominant environmental factors are suitable for simulating the potential distribution of soybean and rapeseed in China, and the simulation results are favorable.

### 3.2. Analysis of suitability for major environmental factors

The quantitative relationship between the logistic probability of the presence and bioclimatic variables was determined through response curves [27]. Among them, the solid red line represents the average value of the results of 10 operations. The greater the probability of existence, the stronger the relationship between the environmental factor value and the species distribution.

**Fig 3:**
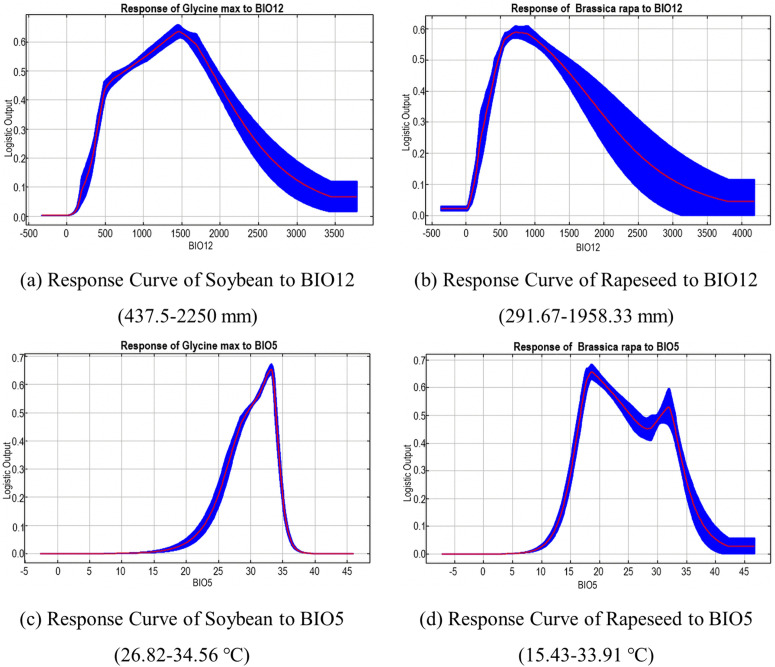
Response Curves of Soybean and Rapeseed to BIO12 and BIO5, respectively.

The screening results of major environmental factors show that the distribution of soybeans and rapeseeds is jointly affected by BIO12 and BIO5. Fig 3 show that the response curves of soybeans and rapeseed to BIO12 and BIO5 are mainly peak-shaped. Soybeans and rapeseeds peak their species suitability when the annual precipitation is about 1458.33 mm and 791.67 mm, and the highest temperature in the hottest month is about 33.18°C and 18.33 °C (the secondary peak of rapeseed is 31.67 °C). It can be seen that both soybeans and rapeseed prefer a warm and humid growing environment. Excessive temperature or precipitation can lead to heat stress and root hypoxia. Compared with soybeans, rapeseed is more adaptable to the environment with less precipitation, and the high temperature tolerance threshold is higher, resulting in a relatively light impact in the sensitive stage.

**Fig 4:**
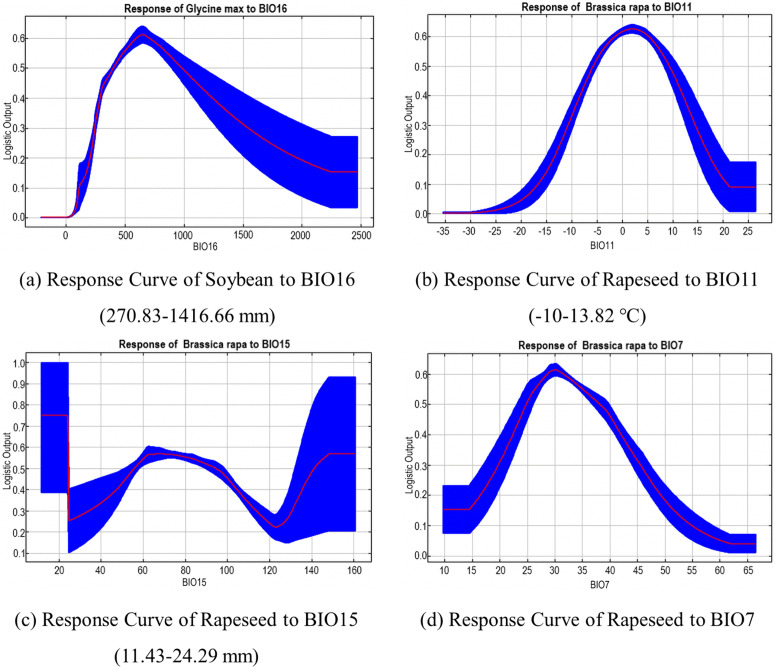
Response Curves of Soybean and Rapeseed to Different Environmental Factors.

In addition to the above factors, soybeans are also significantly affected by BIO16. The response curve to BIO16 (Fig 4a) also shows a single peak; that is, when the precipitation in the wettest season is about 625 mm, the growth suitability is the highest.

Rapeseed is also significantly affected by BIO11, BIO15, and BIO7 (Fig 4 b-d). Rapeseed has a peak value in the coldest season when the average temperature is about 1.88 degrees Celsius, and the annual temperature is about 30 °C, which shows that rapeseed has strong temperature resistance and low sensitivity to temperature. In contrast, rapeseed is more sensitive to the seasonality and stability of precipitation. The response curve of BIO15 (Fig 4c) reflects the highest growth suitability when BIO15 is less than 20.79; that is, the growth is optimal in the area with uniform distribution in the precipitation season.

### 3.3. Potential distribution of soybean and rapeseed in historical and future periods

**Fig 5:**
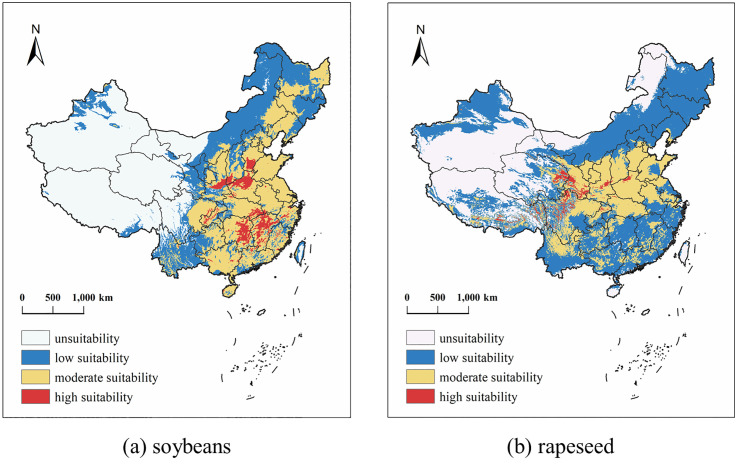
Distribution of Soybeansand Rapeseed in Historical Periods in China. **(Map source: Standard Map Service of the Ministry of Natural Resources, China (Approval NO.:GS(2020)4619), and the base map was not modified. (** **http://bzdt.ch.mnr.gov.cn/** **).**

Historically, the appropriate distribution areas (high and medium suitable areas) of soybeans and rapeseed (Fig 5) in China were mainly concentrated in the central and eastern regions. The suitable area for soybeans is 9.76 × 10^5^ km^2^, more than that of rapeseed, which is more widely distributed in the south and northeast regions and has more advantages in planting. The low-suitable and unsuitable areas of soybeans and rapeseed are mainly distributed in Inner Mongolia and the northwest. However, the planting suitability of rapeseed in Xinjiang, Qinghai, Tibet, and other places is higher than that of soybeans, and the proportion of unsuitable areas of rapeseed is 33.6% lower than that of 44.23% of soybeans.

**Fig 6:**
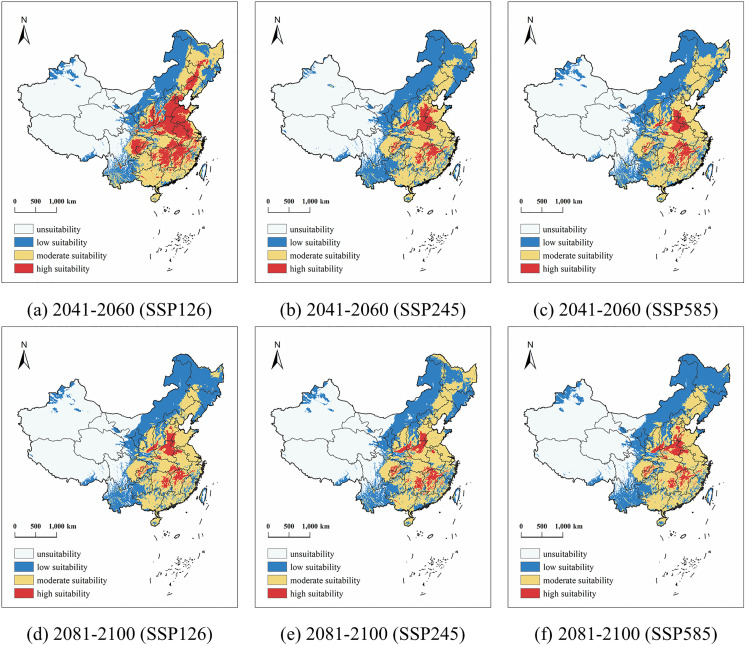
Potential Distribution of Soybean in China under three climate scenarios from 2041 to 2060 and from 2081 to 2100 (Map source: Standard Map Service of the Ministry of Natural Resources, China (Approval NO.:GS(2020)4619), and the base map was not modified. (**http://bzdt.ch.mnr.gov.cn/).**

It can be seen from the future distribution map of soybeans (Fig 6) and the future distribution area table (Table 2) that the future potential distribution of soybeans mainly shows the expansion of the high-suitable areas in the North China Plain and the south of the Yangtze River, the slight shrinkage of the medium-suitable areas in the northeast, and the discrete distribution of the increase in the unsuitable areas in the northwest. The boundary of the high-suitability area will increase the expansion area to the east and north by about 0.14–9.33%, and the increase in the SSP126 scenario in the middle of the 21st century will reach a maximum of 9.17 × 10^5^ m^2^. The driving factor may be the moderate increase in the accumulated temperature, so that the accumulated temperature in the medium and low suitable areas will be transformed into highly suitable areas. The shrinkage of the medium-sized suitable area is about 2.06–5.05%. In the medium term, the Sanjiang Plain under SSP245 shrinks to the south, and the suitability of soybeans may be forced to decline due to high temperatures. The number of low-suitable areas in Xinjiang and other places in the northwest region increased by about 1.26–3.26%, that is, the ecological environment in the northwest region is increasingly unsuitable for crop growth under the high emission scenario. Compared with the three future climate scenarios, the middle of the 21st century is more conducive to the cultivation of soybeans, among which the low-emission scenario is the most significant.

**Table 2:** Potential Distribution Areas of Soybean in China under Three Climate Scenarios during the Historical Period and mid-to-late 21st Century.

Periods	Scenario	Area Suitable for Soybean(×10^4^ km^2^)							
		Unsuitability	Proportion	Low Suitability	Proportion	Moderate Suitability	Proportion	High Suitability	Proportion
Historical Period		434.53	44.23%	246.44	25.09%	271.83	27.67%	29.56	3.01%
2041–2060	SSP126	446.14	45.42%	192.71	19.62%	222.22	22.62%	121.22	12.34%
	SSP245	441.56	44.95%	269.97	27.48%	231.93	23.61%	38.81	3.95%
	SSP585	450.43	45.86%	242.82	24.72%	251.55	25.61%	37.47	3.81%
2081–2100	SSP126	441.74	44.97%	269.73	27.46%	237.92	24.22%	32.90	3.35%
	SSP245	445.71	45.38%	253.10	25.77%	252.54	25.71%	30.93	3.15%
	SSP585	431.09	43.89%	281.83	28.69%	236.40	24.07%	32.95	3.35%

**Fig 7:**
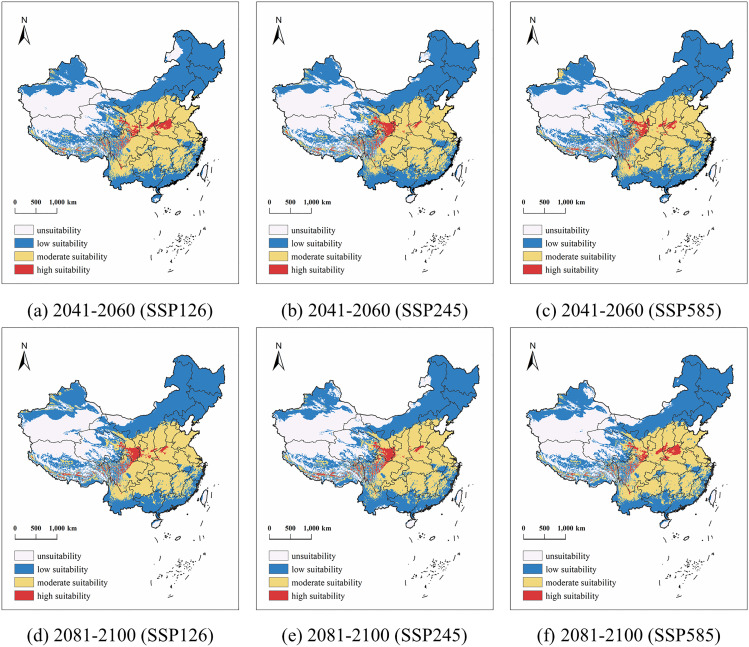
Potential Distribution of Rapeseed in China under three climate scenarios from 2041 to 2060 and from 2081 to 2100 (Map source: Standard Map Service of the Ministry of Natural Resources, China (Approval NO.:GS(2020)4619), and the base map was not modified. (**http://bzdt.ch.mnr.gov.cn/).**

From the future distribution map of rapeseed (Fig 7) and the future distribution area table (Table 3), it can be found that rapeseed will show the discrete distribution pattern of high-suitable areas in the future, the expansion of the hills on the edge of the Sichuan Basin of the Loess Plateau, the rise of medium-suitable areas in the central region, and the shrinkage of unsuitable areas in the northwest and eastern part of Inner Mongolia. The boundary of the high-suitable zone expanded from east to west at the same latitude of about 0.75–0.88%, and the largest expansion of SSP585 in the 2081 period was 0.88%, mainly manifested in the expansion of high-altitude areas such as the Loess Plateau. The overall southward expansion of the medium-suitable area was about 6.23–7.44%, and the maximum increase occurred in the SSP126 scenario in 2041. Areas below low suitability show plaque-like shrinkage, that is, in Xinjiang Oasis and Mengdong, the suitability is improved due to the increase in precipitation. In contrast, rapeseed has a higher adaptability to the hydrothermal conditions of the SSP126 scenario in 2041.

**Table 3:** Potential Distribution Areas of Rapeseed in China under Three Climate Scenarios during the Historical Period and Mid-to-Late 21st Century.

Periods	Scenario	Area Suitable for Oilseed Rape(×10^4^ km^2^)							
		Unsuitability	Proportion	Low Suitability	Proportion	Moderate Suitability	Proportion	High Suitability	Proportion
Historical Period		309.28	32.66%	421.50	44.52%	202.65	21.40%	13.39	1.41%
2041–2060	SSP126	253.49	25.81%	423.42	43.11%	283.27	28.84%	22.09	2.25%
	SSP245	263.06	26.78%	429.58	43.73%	268.38	27.32%	21.24	2.16%
	SSP585	241.84	24.62%	438.73	44.66%	280.02	28.51%	21.72	2.21%
2081–2100	SSP126	233.71	24.68%	420.77	44.44%	271.22	28.64%	21.18	2.24%
	SSP245	265.76	28.07%	398.34	42.07%	261.15	27.58%	21.60	2.28%
	SSP585	244.00	25.77%	415.67	43.90%	265.47	28.04%	21.73	2.29%

In the future, soybeans and rapeseed will generally show a discrete distribution of north-south expansion in suitable areas. The suitable areas for planting of the two overlapped highly, which is the most adaptable to the climate under the low-emission scenario in the 2041 period, with an increase of 4.28% and 8.28%, respectively. However, soybeans are significantly more sensitive to future climate change than rapeseed, and they are susceptible to high temperature stress and precipitation, resulting in a decline in their cultivation advantages.

## 4. Discussion

This study draws the following key findings through the MaxEnt model and GIS spatial analysis, combined with CMIP6 climate scenarios. The high and medium suitable areas of soybeans and rapeseed in China overlap highly, mainly concentrated in the Huang-Huai-Hai region and the Yangtze River Basin. The high-suitability area under the low-emission scenario of the 2041 period greatly expanded to the high latitude of soybeans, far exceeding 11 times that of rapeseed in the same period. In the future, the highly suitable areas for rapeseed will mainly migrate to high latitudes and high altitudes, and the increase will be relatively significant under the high emission scenario of 2081. From the perspective of environmental factors, soybeans are sensitive to the response to annual precipitation and accumulated temperature. It can be seen that the moisture and temperature resistance of soybeans is much weaker than that of rapeseed, and they are more sensitive to future climate change. Rapeseed has a significant response to the average temperature in the coldest season, and its survival is susceptible to winter temperature restrictions.

Soybean rapeseed has a growth habit of being warm and humid, and at the same time, it is limited by the temperature and humidity threshold, so it has a suitable distribution with highly overlapping regions in the mid-latitude region. The highly suitable areas of soybean rapeseed will have different degrees of expansion in the future, which is closely related to the carbon dioxide effect. Studies have shown that high concentrations of carbon dioxide will not only increase the temperature but also have a fertilizer effect, increasing crop biomass and yield, resulting in the expansion of the planting boundary to the north [28,29]. In the future, China’s heat resources will not only expand to the north [30], but also expand into areas with high altitudes in the middle latitude, resulting in an increase in calories in areas with insufficient heat into highly suitable areas for soybean and rapeseed. This is consistent with the conclusion proposed by Feng [31] and others that the suitability of soybeans in the northern hemisphere is significantly improved and that rapeseed shows high altitude expansion. In addition, the sixth IPCC report pointed out that the increase in precipitation in China’s monsoon area in the future will promote the growth of soybean and rapeseed to a certain extent.

However, the positive effects of carbon dioxide and precipitation on crop yields are limited, and may be partially offset by stronger temperature and precipitation increases in the future, resulting in negative interactions [32]. The reason is that the rising temperature will cause frequent heat stress during the growing season of crops, resulting in high-temperature coercion and shortening the nutrient accumulation time [33], resulting in a decrease in yield and a decrease in the suitability of soybeans in Heilongjiang. Excessive precipitation will cause hypoxia to death in the roots of waterlogging-resistant soybeans, or affect the development of rapeseed during the flowering period, and thus affect the yield. Therefore, the growth adaptability of soybean rapeseed under moderate hydrothermal conditions is higher; that is, the high-suitable area of soybeans has a significant increase under the low-emission scenario in the 2041 period, but with the increase of emissions and the passage of time, the increase in the high-suitable area has plummeted to less than 1%. Compared with soybeans, rapeseed is more moisture-resistant and cold-resistant. The limiting factors of rapeseed are moderate in the coldest season, and the precipitation is alleviated in the future. In particular, the precipitation adaptation and the expansion of the accumulated temperature in the 2081 period led to the lifting of the low-temperature restriction, which significantly improved the suitability of rapeseed in the southwestern hilly areas. This is consistent with the conclusion that HE [34] pointed out that the future will be beneficial to rapeseed cultivation in the Yunnan-Guizhou Plateau and Sichuan Basin.

Not only is it distributed in China, but in the future, the potentially suitable areas for global soybeans will expand to high latitudes, while low-latitude areas will have a shrinkage trend due to high temperature coercion or reduced precipitation. This is consistent with the trend of soybeans extending to Ontario and Quebec further north in Canada [35], expanding to high latitudes in southern Brazil [36], and increasing soybean production in Northern Europe while reducing production in southern and southeastern Europe [37]. The migration of global rapeseed to high-altitude areas is more significant than that of soybeans, but the shrinkage in potentially suitable areas in low-latitude areas is relatively small. In the future, the United States will migrate to the high-altitude areas of New Mexico [38] and the rapeseed-friendly areas of Australia to the south-central region [39].

Based on the highly overlapping suitable areas and similar growth habits of soybeans and rapeseed, in order to ensure food security and improve land utilization, reasonable rotation and intercropping can be carried out in combination with regional advantages and the superior varieties of soybean rapeseed. At present, the domestic planting method of soybeans and rapeseed adopts the “early rice-fresh autumn soybeans-rapeseed” system for water and drought rotation, winter rapeseed-corn and soybean intercropping to improve efficiency, and frame soybeans-winter rapeseed-summer soybeans for no-till planting. Previous studies have shown that the preventive effect of rapeseed pre-crop on later rapeseed is more than 80%, indicating that soybean-rapeseed rotation has high ecological and economic benefits [40]. Therefore, it is possible to consider promoting the soybean-rapeseed rotation model in overlapping suitable areas, giving priority to planting soybeans in new suitable areas at high latitudes, and giving priority to the arrangement of rapeseed in new suitable areas on the edge of the Sichuan Basin. In the northwestern region with limited precipitation, agricultural water reserves should be increased to stabilize production [41].

However, the Maxent model has varying suitability and corresponding limitations for different models and crops. Cobben [42] found that the Maxent model is more useful for endemic taxa than for species with broader distributions. The main reason is that the test samples of this model are randomly selected, thus not systematically excluding specific areas, which increases the detection of species distribution in undiscovered regions, leading to overestimated results. Additionally, although the Maxent model has a wider application scope compared to other models and relies only on existing distribution data, it is highly sensitive to certain key parameters. If critical parameters such as the number of background points are neglected or insufficiently discussed, high uncertainty may easily arise. Therefore, when conducting niche analysis for species, to achieve high-accuracy simulation results, one should consider whether their taxonomic group has a relatively concentrated distribution range and use multiple methods to quantify crop sensitivity factors, thereby reducing simulation errors. This study only analyzed dominant climatic factors and failed to fully account for key non-climatic variables, including anthropogenic activities, land use patterns, and soil properties. This oversight may result in model overfitting or overestimated niche outcomes. Future research should incorporate a comprehensive set of influencing factors to enhance the model’s realism and predictive accuracy.

## 5. Conclusions

In the future, climate change will have a significant impact on the potential distribution of soybeans and rapeseed in China. The difference in the response of the two types of crops to climate factors is that soybeans are relatively sensitive, while rapeseed is relatively weakly affected, thus gaining greater growth advantages. However, in the short-term low-emission scenario, soybeans showed a more significant expansion trend. Therefore, the high temperature and precipitation in summer have become limiting factors for soybeans and rapeseed in the future. This kind of conclusion can provide a scientific basis for the layout of the two types of crops in the future. It can effectively meet the challenges brought about by future climate change by cultivating excellent varieties and optimising the planting system, so as to maximize China’s agricultural output.

## Supporting information

S1 Fig Test plot of the importance of climatic factors on the distribution of soybeans during the simulated historical period based on the Jackknife method. (TIF)

S2 Fig Test plot of the importance of climatic factors on the distribution of oilseed rape in the simulated historical period based on the Jackknife method. (TIF)

S1 Table Contributions and replacement importance of climatic factors affecting the distribution of soybeans in China during the historical period. (XLSX)

S2 Table Contributions and replacement importance of climatic factors influencing the distribution of oilseed rape in China during the historical period. (XLSX)

S1 File Dominant environmental factor screening. (DOCX)

## References

[1] Rockström J, Edenhofer O, Gaertner J, DeClerck F. Planet-proofing the global food system. Nat Food. 2020;1(1):3–5. doi: 10.1038/s43016-019-0010-4

[2] Liu T, Yang X, Batchelor WD, Liu Z, Zhang Z, Wan N, et al. A case study of climate-smart management in foxtail millet (Setaria italica) production under future climate change in Lishu county of Jilin, China. Agricultural and Forest Meteorology. 2020;292–293:108131. doi: 10.1016/j.agrformet.2020.108131

[3] Bao L, Yu L, Li Y, Yan F, Lyne V, Ren C. Climate change impacts on agroecosystems in China: Processes, mechanisms and prospects. Chin Geogr Sci. 2023;33(4):583–600. doi: 10.1007/s11769-023-1362-0

[4] Wu S, Wu Y, Wen J. Future changes in precipitation characteristics in China. Intl Journal of Climatology. 2019;39(8):3558–73. doi: 10.1002/joc.6038

[5] Zhou S, Zhou W, Lin G, Chen J, Jiang T, Li M. Adapting to climate change: scenario analysis of grain production in China. CAER. 2017;9(4):643–59. doi: 10.1108/caer-10-2016-0173

[6] Li R, Geng S. Impacts of climate change on agriculture and adaptive strategies in China. Journal of Integrative Agriculture. 2013;12(8):1402–8. doi: 10.1016/s2095-3119(13)60552-3

[7] Li X, Zhang L, Chen N, Huang Y, Tan F, Li S, et al. Potential dynamic changes of single-season rice planting suitability across China. Int J Biometeorol. 2023;67(5):875–86. doi: 10.1007/s00484-023-02462-y 37010576

[8] Zhang Y, Wang Y, Niu H. Spatio-temporal variations in the areas suitable for the cultivation of rice and maize in China under future climate scenarios. Sci Total Environ. 2017;601–602:518–31. doi: 10.1016/j.scitotenv.2017.05.232 28575830

[9] Yang J, Hu Q, You L, Cai Z, Chen Y, Wei H, et al. Mapping the potential northern limits and promotion extent of ratoon rice in China. Applied Geography. 2023;150:102822. doi: 10.1016/j.apgeog.2022.102822

[10] Zhao C, Zhang F, Huang J, Zhang Q, Lu Y, Cao W. Prediction of the climatically suitable areas of rice in China based on optimized MaxEnt Model. Int J Plant Prod. 2024;18(4):549–61. doi: 10.1007/s42106-024-00309-z

[11] Guo X, Zhang P, Yue Y. Prediction of global wheat cultivation distribution under climate change and socioeconomic development. Sci Total Environ. 2024;919:170481. doi: 10.1016/j.scitotenv.2024.170481 38307262

[12] Yue Y, Zhang P, Shang Y. The potential global distribution and dynamics of wheat under multiple climate change scenarios. Sci Total Environ. 2019;688:1308–18. doi: 10.1016/j.scitotenv.2019.06.153 31726560

[13] He X. Comparative study on the competitiveness of soybeans from the perspective of technological progress. Hebei Academic Journal. 2024;44(03):135–46.

[14] Tang X, Liu H. Climate suitability for summer maize on the North China Plain under current and future climate scenarios. Intl Journal of Climatology. 2020;41(S1). doi: 10.1002/joc.6872

[15] Chen Y, Ren L, Lou Y, Tang L, Yang J, Su L. Effects of climate change on climate suitability of green orange planting in Hainan Island, China. Agriculture. 2022;12(3):349. doi: 10.3390/agriculture12030349

[16] Zhang Z, Yan Y, Tian Y, Li J, He J-S, Tang Z. Distribution and conservation of orchid species richness in China. Biological Conservation. 2015;181:64–72. doi: 10.1016/j.biocon.2014.10.026

[17] Tang S-L, Song Y-B, Zeng B, Dong M. Potential distribution of the extremely endangered species Ostrya rehderiana (Betulaceae) in China under future climate change. Environ Sci Pollut Res Int. 2022;29(5):7782–92. doi: 10.1007/s11356-021-16268-1 34476707

[18] Ruijiang W, Xin W. Progress and prospects of research on climatic suitability at home and abroad. Advances in Earth Science. 2019;34(6):584.

[19] Jaynes ET. Probability theory: The logic of science. Cambridge University Press. 2003.

[20] Golan A, Foley DK. Understanding the constraints in maximum entropy methods for modeling and inference. IEEE Trans Pattern Anal Mach Intell. 2023;45(3):3994–8. doi: 10.1109/TPAMI.2022.3185394 35737619

[21] Soilhi Z, Sayari N, Benalouache N, Mekki M. Predicting current and future distributions of Mentha pulegium L. in Tunisia under climate change conditions, using the MaxEnt model. Ecological Informatics. 2022;68:101533. doi: 10.1016/j.ecoinf.2021.101533

[22] Zhao Y, Wen Y, Zhang W, Wang C, Yan Y, Hao S, et al. Distribution pattern and change prediction of Phellodendron habitat in China under climate change. Ecol Evol. 2023;13(8):e10374. doi: 10.1002/ece3.10374 37636866 PMC10450841

[23] O’Neill BC, Kriegler E, Riahi K, Ebi KL, Hallegatte S, Carter TR, et al. A new scenario framework for climate change research: The concept of shared socioeconomic pathways. Climatic Change. 2013;122(3):387–400. doi: 10.1007/s10584-013-0905-2

[24] Hosni EM, Nasser MG, Al-Ashaal SA, Rady MH, Kenawy MA. Modeling current and future global distribution of Chrysomya bezziana under changing climate. Sci Rep. 2020;10(1):4947. doi: 10.1038/s41598-020-61962-8 32188920 PMC7080715

[25] Zhao Y, Zhao M, Zhang L, Wang C, Xu Y. Predicting Possible Distribution of Tea (Camellia sinensis L.) under Climate Change Scenarios Using MaxEnt Model in China. Agriculture. 2021;11(11):1122. doi: 10.3390/agriculture11111122

[26] Xian Y, Liu G, Yao H. Predicting the current and future distributions of major food crop designated geographical indications (GIs) in China under climate change. Geocarto International. 2021;37(25):8148–71. doi: 10.1080/10106049.2021.1993352

[27] Jayasinghe SL, Kumar L. Modeling the climate suitability of tea [Camellia sinensis(L.) O. Kuntze] in Sri Lanka in response to current and future climate change scenarios. Agricultural and Forest Meteorology. 2019;272–273:102–17. doi: 10.1016/j.agrformet.2019.03.025

[28] Lenka NK, Lenka S, Thakur JK, Yashona DS, Shukla AK, Elanchezhian R, et al. Carbon dioxide and temperature elevation effects on crop evapotranspiration and water use efficiency in soybean as affected by different nitrogen levels. Agricultural Water Management. 2020;230:105936. doi: 10.1016/j.agwat.2019.105936

[29] Yang X, Chen F, Lin X, Liu Z, Zhang H, Zhao J, et al. Potential benefits of climate change for crop productivity in China. Agricultural and Forest Meteorology. 2015;208:76–84. doi: 10.1016/j.agrformet.2015.04.024

[30] Chu Z, Guo J, Zhao J. Impacts of future climate change on agroclimatic resources in Northeast China. J Geogr Sci. 2017;27(9):1044–58. doi: 10.1007/s11442-017-1420-6

[31] Feng L, Wang H, Ma X, Peng H, Shan J. Modeling the current land suitability and future dynamics of global soybean cultivation under climate change scenarios. Field Crops Research. 2021;263:108069. doi: 10.1016/j.fcr.2021.108069

[32] Makowski D, Marajo-Petitzon E, Durand J-L, Ben-Ari T. Quantitative synthesis of temperature, CO2, rainfall, and adaptation effects on global crop yields. European Journal of Agronomy. 2020;115:126041. doi: 10.1016/j.eja.2020.126041

[33] Zheng H, Zhang L, Sun H, Zheng A, Harrison MT, Li W, et al. Optimal sowing time to adapt soybean production to global warming with different cultivars in the Huanghuaihai Farming Region of China. Field Crops Research. 2024;312:109386. doi: 10.1016/j.fcr.2024.109386

[34] He Y. Study on the Climate Change Impact on Chinese Oilseed Rape Production: Huazhong Agricultural University; 2016.

[35] Qian B, Smith W, Jing Q, Kim YM, Jégo G, Grant B, et al. Climate conditions in the near-term, mid-term and distant future for growing soybeans in Canada. Can J Plant Sci. 2023;103(2):161–74. doi: 10.1139/cjps-2022-0233

[36] Figueiredo Moura da Silva EH, Silva Antolin LA, Zanon AJ, Soares Andrade A Junior, Antunes de Souza H, dos Santos Carvalho K, et al. Impact assessment of soybean yield and water productivity in Brazil due to climate change. European Journal of Agronomy. 2021;129:126329. doi: 10.1016/j.eja.2021.126329

[37] Marteau-Bazouni M, Jeuffroy M-H, Guilpart N. Grain legume response to future climate and adaptation strategies in Europe: A review of simulation studies. European Journal of Agronomy. 2024;153:127056. doi: 10.1016/j.eja.2023.127056

[38] Djaman K, O’Neill M, Owen C, Smeal D, West M, Begay D, et al. Seed yield and water productivity of irrigated winter canola (Brassica napus L.) under Semiarid Climate and High Elevation. Agronomy. 2018;8(6):90. doi: 10.3390/agronomy8060090

[39] Xing H, Brill R, Li G, Li L, Blake A, Slinger D. Future climate change increases canola productivity and water use efficiency in the rainfed cropping systems of Southern Australia. 19th Australian Agronomy Conference 2019; 2019: Australian Society of Agronomy.

[40] Yang XX, Zhang L, Huang XQ, Wu WX, Zhou XQ, Du L, et al. Difference of the microbial community structure in the rhizosphere of soybean and oilseed rape based on high-throughput pyrosequencing analysis. Ying Yong Sheng Tai Xue Bao. 2019;30(7):2345–51. doi: 10.13287/j.1001-9332.201907.028 31418238

[41] Zhu Q, Wang F, Yi Q, Zhang X, Chen S, Zheng J, et al. Modeling soybean cultivation suitability in China and its future trends in climate change scenarios. J Environ Manage. 2023;345:118934. doi: 10.1016/j.jenvman.2023.118934 37690252

[42] Cobben MMP, van Treuren R, Castañeda-Álvarez NP, Khoury CK, Kik C, van Hintum TJL. Robustness and accuracy of Maxent niche modelling for Lactuca species distributions in light of collecting expeditions. Plant Genet Resour. 2014;13(2):153–61. doi: 10.1017/s1479262114000847

