# Supplementary material for: Analyzing the distribution patterns of soybeans and rapeseeds in china under future climate change scenarios utilizing the maxent model
Source: PLoS One. 2026 Apr 1;21(4):e0342400. doi: 10.1371/journal.pone.0342400 (PMC13042770; doi:10.1371/journal.pone.0342400)
Supplement: S1 File — (DOCX) [file pone.0342400.s005.docx]

**Dominant environmental factor screening**

Model simulations based on 19 bioclimatic factors from historical periods revealed the importance of each factor in affecting the distribution of soybean and rapeseed. The colored strips in the regularization training gain display represent the contribution of each variable to the distribution potential of soybeans and rapeseeds. The longer the blue strip is, the greater the contribution of the corresponding factor is^[1]^. In combination with the contribution rate table, select the factors with a cumulative contribution rate of more than 68% and a contribution rate of less than 5% to the last variable, and then compare them with the correlation analysis results of ENMTools, so as to determine the dominant factors.

As shown in S1 Fig., among the bioclimatic factors affecting soybeans, the top eight factors ranked from high to low importance are BIO10, BIO5, BIO1, BIO8, BIO12, BIO2, BIO13, and BIO16. According to S3 Table., the cumulative contribution rate of BIO10, BIO13, BIO12, BIO4, and BIO5 has reached 68.4%, exceeding 68%, indicating that these factors have a significant impact on the results. Although the contribution rate of BIO16 is low, its replacement importance is 15%, so it is also an important factor affecting the results. If the contribution degree, replacement importance, and training gain value of BIO4 are relatively low, the simulation effect can be ignored. Even if the gain values of BIO10 and BIO13 are greater than 0.5, their replacement importance is less than 5, and they are collinear with BIO5 and BIO12, respectively, indicating a weak independent ecological effect. The dominant environmental factors affecting the soybean distribution in China were BIO12 (annual precipitation), BIO5 (max temperature in the warmest month), and BIO16 (precipitation in the wettest quarter).

Regarding the dominant environmental factors of rapeseed, according to the importance test chart (S2 Fig.) using the Jackknife method, the factors affecting its potential distribution are arranged according to the importance levels BIO11, BIO6, BIO12, BIO9, BIO16, BIO18, BIO7, and BIO. Eight. Among them, the blue bar of BIO11 is large, and the gain training value is greater than 0.3, which plays a key role in determining the appropriate distribution of rapeseed. In addition, based on the results of S4 Table., the cumulative contribution rates of BIO12, BIO11, BIO5, BIO15, BIO7, and BIO4 reached 68.9%. Among them, the total contribution rate of BIO12 and BIO11 is 46.6%, indicating that these variables contain more predictive information. In contrast, the contribution rate and arrangement importance of BIO6 and other factors are low, indicating that their impact on the distribution of rapeseed is minimal and negligible. The correlation index of BIO4 and BIO7 is 0.97, which is relatively high, so the impact of BIO4 on the distribution of rapeseed can be ignored. The dominant climatic factors affecting the oilseed rape distribution in China were BIO12 (annual precipitation), BIO11 (mean temperature in the coldest quarter), BIO5 (max temperature in the warmest month), BIO15 (precipitation seasonality), and BIO7 (temperature annual range), with BIO12 and BIO11 being the most dominant environmental factors.

**References**

1. Gao H, Qian Q, Deng X, et al. Predicting the Distributions of Morus notabilis CK Schneid under Climate Change in China[J]. Forests, 2024, 15(2): 352.
